# Effects and safety of Ophiocordyceps sinensis preparation in the adjuvant treatment for dialysis patients: A protocol for systematic review and meta-analysis

**DOI:** 10.1097/MD.0000000000031476

**Published:** 2022-11-18

**Authors:** Meixi Liu, Tianying Chang, Di Zou, ChengJi Cui, Chunyan Liu, Shoulin Zhang, Xing Liao

**Affiliations:** a Changchun University of Chinese Medicine, Changchun, China; b Evidence-based Medicine Office, the Affiliated Hospital to Changchun University of Chinese Medicine, Changchun, China; c Nephropathy Department, the Affiliated Hospital to Changchun University of Chinese Medicine, Changchun, China; d Institute of Clinical Basic Medicine of Chinese Medicine, Academy of Chinese Medical Sciences, Beijing, China.

**Keywords:** dialysis, end stage renal disease(ESRD), Ophiocordyceps sinensis preparation, protocol

## Abstract

*Ophiocordyceps sinensis*(*O. sinensis*), a genus of ascomycete fungi, has been intensively studied in various disease models, which is a rich source of various bioactive compounds and used in the treatment for end-stage renal disease patients. This systematic review highlights the therapeutic roles of *O. sinensis* as adjuvant treatment for dialysis patients with clinical evidence.

**Methods and analysis:**

The systematic review will be performed according to the Cochrane Handbook for Systematic Reviews of Interventions. The protocol is being reported in accordance with the Preferred Reporting Items for Systematic Review and Meta-Analysis Protocols Statement. An literature search strategy will be developed and adapted for 9 databases. Searches will be run from the database inception until the date of the search implementation and be updated before the review is completed. Randomized controlled trials that investigate the effects of O. sinensis for dialysis patients (peritoneal dialysis and hemodialysis) will be included. We will focus on outcomes recommended by the core outcome measures in effectiveness trials, including mortality, cardiovascular disease, infection, vascular access problems, dialysis adequacy, hyperkalaemia, life participation. Two researchers will independently screen the studies, extract data and evaluate study quality using the Risk of Bias 2 tool. Subgroup analysis will be performed according to peritoneal dialysis and hemodialysis. Sensitivity analyses will be conducted based on the Leave-1-Out Method. The Grading of Recommendations Assessment, Development, and Evaluation approach will be used to rate the quality of the evidence. Meta analysis will be performed using Review Manager 5.3 and R packages.

**Objectives:**

Studies have reported positive results of O. sinensis as adjuvant treatment for patients with dialysis. This review will synthesis current evidence on how O. sinensis can improve dialysis. Thus, it is expected that robust and conclusive evidence of the effects of O. sinensis during or after treatment can be obtained. These findings can inform future research and the selection of O. sinensis to promote quality of life for people with dialysis.

## 1. Introduction

Dialysis is a treatment that removes wastes and extra fluid from patient blood when patient’s kidneys are no longer able to work effectively.^[1]^ Patients need dialysis when they develop end stage kidney failure, usually by the time they lose about 85% to 90% of their kidney function and have a glomerular filtration rate that falls below 15 mL/minute/1.73m^2^.^[2]^ There are 2 types of dialysis procedures, of which 1 is hemodialysis using a machine/artificial kidney-like apparatus, the other is peritoneal dialysis using a peritoneal membrane as a filter. Hemodialysis is done for patients with no residual renal function where as peritoneal dialysis is recommended for younger patients due to its flexibility. In chronic or end stage kidney failure, dialysis is the best method to remove accumulated toxins from the body and improve the quality of life for the rest of life. However individuals suffering from chronic renal failure, who are on dialysis, could be at increased cardiovascular and metabolic risk and an increased risk for getting an infection.^[3]^ Dialysis vintage is associated with an enhanced risk of death, with each additional year of dialysis treatment associated with an increase in the risk of death of approximately 6%.^[4]^ Based on the United States Renal Data System (USRDS) report, the adjusted survival rate for patients on hemodialysis (HD) is 57% at 3 years after onset of end stage renal disease as compared to 68% for patients receiving peritoneal dialysis (PD). The 5-years survival for patients receiving HD and PD is 42% and 52%, respectively.^[5]^

Among patients with maintenance dialysis, the mortality rate is high at about 165/1000.^[6]^ Many patients develop malnutrition and a micro-inflammatory state due to tubing during dialysis, reduced food intake and intestinal digestion and absorption, and metabolic acidosis.^[7,8]^ Numerous complications also affect patients’ quality of life and increase mortality. Therefore, improving the complications is very important for prolonging the lifespan of patients and improving their quality of life.

*Ophiocordyceps sinensis* (*O. sinensis*), also named Chinese caterpillar fungus, is a precious traditional medicine which is mostly distributed on the Qinghai-Tibetan Plateau.^[9]^ For its medicinal values on anti-fatigue, anti-tumor, and kidney protection,^[10–12]^ it has become 1 of the most valuable biological commodities and widely traded in recent years worldwide Modern pharmacological studies found that its main components include *cordyceps* polysaccharide, *cordycepin*, *cordycepic* acid, etc.^[13,14]^ Due to the increasing of vulnerability and risk for the wild *O. sinensis* (overexploitation and habitat loss)^[9]^ and its surged price^[15]^ *O. sinensis* preparations have emerged with a right price. Synthetic *Ophiocordyceps sinensis* preparation is cultivated from strains extracted from *Ophiocordyceps sinensis*.^[16]^ Studies have shown that synthetic *Ophiocordyceps sinensis* preparations can benefit dialysis patients by improving their quality of life, reducing the incidence of cardiovascular events, improving the micro-inflammatory state and malnutrition, etc.^[17–19]^ Although there is a systematic review published in 2019 to evaluate the efficacy of *Cordyceps* sinensis as an adjunctive treatment in hemodialysis patients,^[20]^ we aim to conduct a comprehensive systematic and meta-analysis to evaluate the efficacy and safety of *O. sinensis* preparation in the treatment of both hemodialysis as well as peritoneal dialysis.

## 2. Objectives

This systematic review aims to clarify whether *O. sinensis* preparation in the adjuvant treatment for both hemodialysis (HD) patients and peritoneal dialysis (PD) patients is more effective than the control in reducing cardiovascular disease (CVD) mortality and infection. Our secondary objective is to answer the following 2 questions:

a. Does response to treatment with *O. sinensis* preparation depend on the duration of treatment?
b. What are the side effects of the combined use of *O. sinensis* preparation?

## 3. Methods and analysis

### 3.1. Registration

We drafted the protocol according to the Preferred Reporting Items for Systematic Reviews and Meta-Analysis Protocol (PRISMA-P)^[21]^ (see Table S1, Supplemental Digital Content, http://links.lww.com/MD/H791, which illustrates the PRISMA-P 2015 checklist) and will report this systematic review in adherence to the Preferred Reporting Items for Systematic Reviews and Meta-Analyses(PRISMA).^[22]^ The review has been registered in the international prospective register of systematic reviews (PROSPERO) with the identifier CRD42022324508.

### 3.2. Eligibility criteria

The eligibility criteria has been set based on the PICOS principle: P(participant), I(intervention), C(comparator), O(outcome) and S(study design) as following:

#### 3.2.1. *Type of study.*

Only randomized controlled trials, regardless of whether they are blinded or design or not, that were published in English or Chinese, in peer-reviewed journals will be included in this review.

#### 3.2.2. *Participant.*

Adult participants aged ≥ 18 years, receiving hemodialysis or peritoneal dialysis, regardless of their primary disease, race, gender and ethnicity.

#### 3.2.3. *Intervention.*

*O. sinensis* preparations were taken orally combined with dialysis treatment and conventional treatment. There is no restriction on the specific dosage form, administration, course, and manufacturers. As we know, there are 11 kinds of *O. sinensis* preparations commonly used to treat patients undergoing dialysis, including Bailing Tablets (capsules), Jinshuibao Tablets (capsules), Zhiling Capsules, *Cordyceps* militaris capsules, *Cordyceps* militaris powder, Cultured *Cordyceps sinensis* powder, Powdered *Cordyceps* mortierella mycelia, *Cordyceps* cephalosporium mycelia and Fermentative *Cordycepis* fungal powder. All of them were approved by National Medical Products Administration in mainland China. We would exclude studies if any treatment group and the control group used other traditional Chinese medicine treatments, including Chinese patent medicine and acupuncture.

#### 3.2.4. *Comparator.*

The control group received the same dialysis treatment and conventional treatment with the experimental group. Conventional treatments include low purine, low salt, low fat, low phosphorus quality, a low-protein diet, limited water intake, control of blood pressure, blood lipids, blood sugar, and the symptomatic treatment for the complications.

#### 3.2.5. *Type of outcomes.*

After searching the Core Outcome Measures in Effectiveness Trials (https://www.cometinitiative.org/) – Core outcome measures in effectiveness trials, we uptake the outcomes from the SONG-HD and SONG-PD core outcome sets (COS)^[23,24]^ which was developed by the Standardised Outcomes in Nephrology-Peritoneal Dialysis (Nephrology-Hemodialysis) initiative. Some of the outcomes will be selected for the current review, which are divided into primary outcomes (e.g., mortality, CVD, infection) and secondary outcomes (e.g., vascular access problems, dialysis adequacy, hyperkalaemia, life participation).

### 3.3. Search strategy

A search strategy has been created with the help from an experienced librarian and adapted for searching the databases PubMed, EMBASE, the Cochrane Library, SinoMed, CNKI, VIP, Wanfang Data and International Clinical Trials Register (ICTRP) Search Portal and Clinical Trials.gov to identify RCTs involving the above-mentioned interventions. We will conduct the literature searching from the inception of all the databases to October, 2022 and will update it before the review is completed. Screening reference lists and consulting experts will be performed in this time frame. Studies in accordance with the PICOS will be considered. Key search terms (MeSH and Free words) used for our searches are “Renal Dialysis” or “*Ophiocordyceps sinensis*” and “RCTs” or “*Cordyceps*,” etc. (see Table S1, Supplemental Digital Content, http://links.lww.com/MD/H792, which illustrates the detailed search strategy for all databases).

### 3.4. Study selection

**Figure 1.**
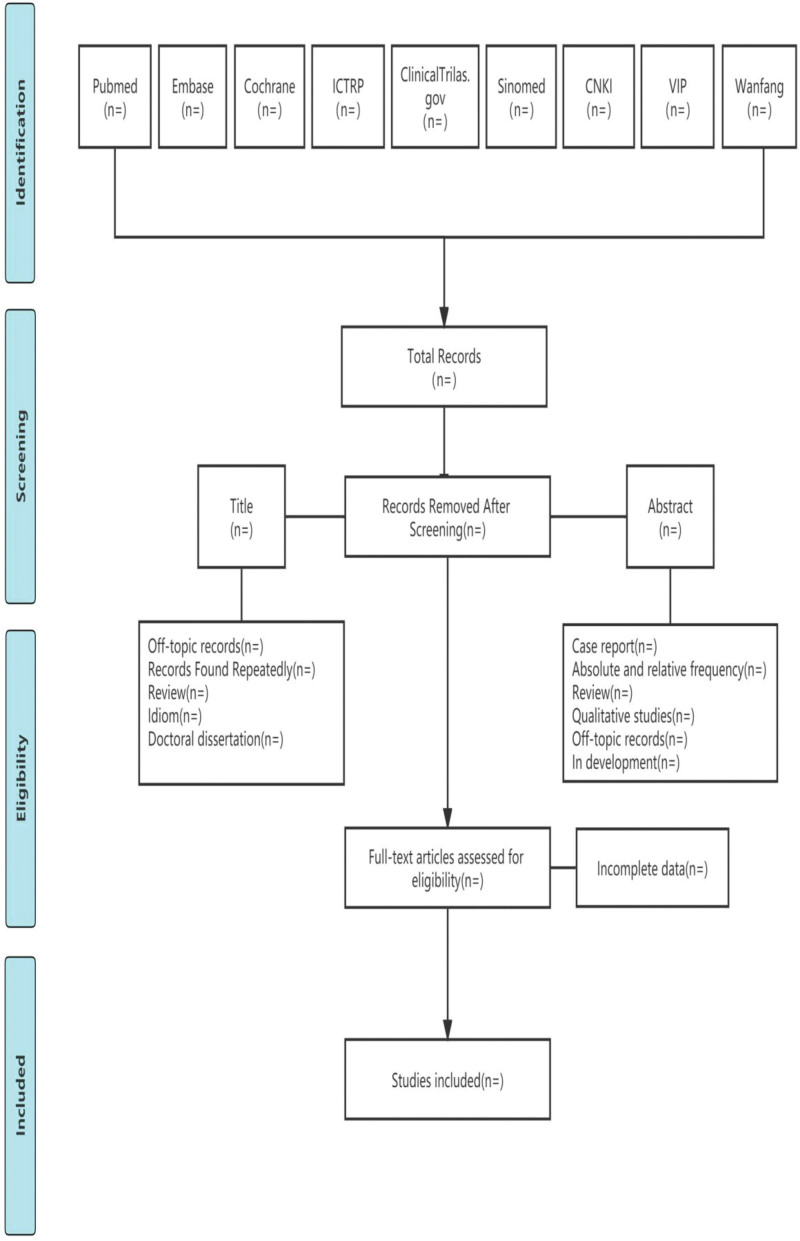
PRISMA flow diagram of the study selecting process.

All retrieved records will be imported into Endnote X9.1 software and the duplicated records will be removed. By referring to the eligibility criteria 2 researchers (MXL and DZ) will independently: screen the title and abstract of deduplicated articles and remove those that do not meet the eligibility criteria; then recheck the full text of the remaining articles and finally include or exclude them. A third reviewer (XL) will be consulted, in case of disagreement. All excluded literature during the full text checking will be recorded and tabulated with their justification for exclusion. The selecting process will follow the PRISMA flow diagram,^[21]^ see Figure 1.

### 3.5. Data extraction

We will extract information from the included studies, and 2 researchers(MXL and DZ) will fill the extracted data in a pre-designed form designed using Excel spreadsheet 2019. The data extraction table will include information as following (Tables 1 and 2):

**Table 1:** Basic characteristics of the included studies.

Study	Author	Year of publication	Country	Language	M/W	Sample size	Average age	Study period	follow-up period
									

**Table 2:** Basic clinical characteristics of the included studies.

Study	Therapy method	Average duration of disease	Mean history of dialysis	Comorbi-dity	Dialysis time	Dialysis frequency	Mortality	CVD	Infection	Vascular access problems	Dialysis adequacy	Hyperkalaemia	Life participa-tion
													

CVD = cardiovascular disease.

1. Study characteristics: published title, author name, journal name, the country where the study was conducted, year of publication, language, sample size, study design, study period, and follow-up period.
2. Participants: male-female ratio, average age, primary disease, disease stage, severity, the average duration of disease, and the mean of the dialysis.
3. Interventions: hemodialysis or peritoneal dialysis; dialysis time, frequency, duration; comorbidity.
4. Outcomes: Primary outcomes: mortality, CVD, infection; Secondary outcomes: vascular access problems, dialysis adequacy, hyperkalaemia, life participation.

Two researchers (MXL and DZ) will independently extract data from all articles that meet the inclusion criteria. All results will be cross-examined. If the cross-examination results are inconsistent, the discussion will resolve the disagreement until consensus is reached or by consulting a third author (TYC). For the data partially mentioned but not shown, we will contact the author by phone or email to obtain it.

### 3.6. Assessing risk of bias

Two researchers (MXL and DZ) will assess the risk of bias for included articles independently according to the Cochrane risk of bias (ROB) tool for interventions.^[25]^ ROB includes 7 domains on which biases within trials are assessed: sequence generation; allocation concealment; blinding of participants and personnel; blinding of outcome assessment; incomplete outcome data; selective reporting and other bias(baseline imbalance between groups of participants, blocked randomization in trials that are not blinded, differential diagnostic activity, etc.). Each domain will be rated as “high,” “unclear” or “low” risk of bias, and are reported separately. The assessment will be graphed and use Review Manager 5.3 software (Cochrane, Northern Europe).

### 3.7. Method for data synthesis

Qualitative evidence synthesis will be performed based on the available results. After describing the baseline characteristics of the studies, the outcome of interest will be summarized, that is, the effects of *O. sinensis* preparations in the adjuvant treatment for both hemodialysis (HD) patients and peritoneal dialysis (PD) patients. Furthermore, the effect evaluation for HD patients and PD patients will be assessed separately. Statistical significance will be set at *P* < .05.

#### 3.7.1. *Meta-analysis.*

A meta-analysis will be carried out if the number of RCTs corresponds to the same PICOS in 2 or more. Effect sizes will be calculated as either RR/OR/HR (for dichotomous data) and weighted (or standardized) final post-intervention mean differences (for continuous data) with their corresponding 95% confidence intervals. Review Manager 5.3 software^[26]^ will be used to conduct meta analyses. The effects models (fixed or random) will be used to estimate the effect of *O. sinensis* preparation by creating forest plots. When heterogeneity is present, the random-effect model will be used. If meta analyses are not appropriate, a narrative description including tables and figures will be performed to summarize *O. sinensis* preparation’s application in the treatment for the dialysis patients and if there were any gaps in the literature.

#### 3.7.2. *Heterogeneity assessment.*

We will estimate the between-study heterogeneity in all of the eligible comparisons, using the Chi^2^-based Q statistic^[27]^ and assess the extent of heterogeneity with *I*^2^, a quantitative measure of inconsistency between studies. When the values are 0% or ≥ 50%, they represent no heterogeneity and considerable levels of heterogeneity, respectively.^[28]^ If the heterogeneity is within the acceptable range, the fixed-effects model will be used to affect estimates; Otherwise, the random-effects model will be used.

#### 3.7.3. *Publication bias.*

We will assess publication bias using funnel plots and Egger tests only when there are at least 10 studies included in the meta-analysis.^[29]^ If the funnel plot shows asymmetry, it will indicate publication bias. If publication bias exists, trim and fill analyses will be used to assess the impact of publication bias on the results. Any bias will be explained through the analyses and discussions.

#### 3.7.4. *Sensitivity analysis.*

Sensitivity analysis will be performed to verify the results’ robustness. We will do this through the leave-1-out strategy^[30,31]^ based on the quality of the included studies to explore the sources of heterogeneity. Namely 1 study is excluded at a time and the impact of removing each of the studies is evaluated on the summary results and the between-study heterogeneity.

#### 3.7.5. *Subgroups analysis.*

Subgroup analysis will be conducted to analyze the causes of heterogeneity. We will do this based on the 2 dialysis modalities (hemodialysis and peritoneal dialysis), sample size, treatment course, and follow-up period.

### 3.8. Quality of the evidence

The certainty of evidence will be graded for each outcome, from a rating of HIGH to VERY LOW by following the grading of recommendations assessment, development, and evaluation (GRADE) approach.^[32]^ The GRADE system includes 5 domains that can downgrade the quality of the evidence used for RCTs-limitations, inconsistent results, imprecision, indirectness, and publication bias. The quality of evidence for each outcome is graded as HIGH, MODERATE, LOW or VERY LOW. A summary of Findings (SoF) will be created using GRADE Pro GDT 2021 (McMaster University, ON, Canada). The SoF will present the following information where appropriate: absolute risks for the treatment and control, estimates of relative risk, and a ranking of the quality of the evidence based on the risk of bias, directness, heterogeneity, precision, and risk of publication bias of the review results. The outcomes reported in the SoF table for this review will be: CVD, mortality, dialysis adequacy, infection, etc.

## 4. Discussion

From 1990 to 2017, with the development of dialysis technology, the incidence of dialysis increased by 43.1%.^[33]^ In the worldwide, approximately 89% of dialysis patients are treated with hemodialysis, and a minority with peritoneal dialysis.^[34]^ The global dialysis population is grow-ing rapidly, especially in low-income and middle-income countries; however, worldwide, a sub-stantial number of people lack access to kidney replacement therapy, and millions of people die of kidney failure annually, often without supportive care.^[35]^ Thus, there is an urgent need to develop new approaches and dialysis modalities that are accessible and offer improved patient outcomes.

*Sinensis,or* Chinese caterpillar fungus or Dong Chong Xia Cao (winter worm-summer grass) in Chinese or Tochukaso in Japanese, has been used in China for > 700 years, mainly as a tonic for nourishing the lungs and nourishing the kidneys.^[36]^ Modern pharmacological studies have shown that it has a therapeutic effect on a variety of diseases and conditions such as the kidneys^[37,38]^ as well as other diseases.^[39]^ However, due to the scarcity of the resource and the high price, the output of natural *O. sinensis* is unable to fully meet the demands of medical use, which drives many types of artificial cultivation to make *O. sinensis* a more affordable material for its use.^[40–42]^ The highest *cordycepin* production now can be obtained in surface liquid culture using the C. militaris mutant^[43]^ The artificial cultivation of C. militaris produces *cordycepin* and has similar pharmacological activity to *O. sinensis*, which is easier and was successfully achieved and multiproduct batch manufacturing has also been achieved.^[44]^ This review aims to assess the effects of *O. sinensis* preparation in the adjuvant treatment for both hemodialysis (HD) patients and peritoneal dialysis (PD) patients. Specifically, the effects of *O. sinensis* preparation on mortality, CVD, infection, vascular access problems, dialysis adequacy, hyperkalaemia, life participation will be analyzed. Thus, we can obtain robust and conclusive evidence regarding the effects of *O. sinensis* preparation to support clinical practice, in addition to contributing high-quality studies on the subject.

To our knowledge, this review will be the first systematic review to evaluate the efficacy and safety of *O. sinensis* preparation in the adjuvant treatment for 2 kinds of dialysis patients (hemodialysis and peritoneal dialysis). Although a systematic review and meta-analysis of hemodialysis patients was published in 2019,^[20]^ our study has many differences from this review. Firstly, our research covers a wider range of subjects, including not only patients with HD but also with PD. Secondly, a (COS) is the minimum that should be measured and reported in all clinical trials of a specific condition,^[45]^ which would also help with streamlining the systematic reviewing process.^[46]^ Therefore, we searched the core outcome measures in effectiveness trials (*Core Outcome Measures in Effectiveness Trials*) Database and referred to the dialysis-COS to set the primary and secondary outcomes for our review. Thirdly, we will compare the patients with hemodialysis dialysis to the patients with peritoneal dialysis in the response to the *O. sinensis*.^[47,48]^ Altogether, it has been 6 years since the literature searching in the last systematic review, and new research evidence has increased. Accordingly, new clinical research problems with different Population, Intervention, Comparison, Outcomes and Study (PICOS) emerged, which urged us to carry out a new systematic review. We hope that this systematic review can provide evidence of efficacy and safety for dialysis patients when using *O. sinensis* preparation.

The strengths of our study could be strict adherence to the PRISMA-P and PRISMA guidelines and the use of a combination of quantitative and qualitative analyses.

However, this systematic review may be limited if there is a scarcity of appropriate studies in literature, heterogeneity of intervention protocols, methodological quality of the studies and absence of data from studies for analysis. We plan to perform narrative synthesis if meta analysis is not possible or appropriate. Subgroup analysis based on pre-specified subgroups where possible and sensitivity analysis will be conducted to explore the heterogeneity. We will contact authors to share with us the missing data if it is necessary.

## Acknowledgments

We acknowledge the Department of Nephrology and the Evidence-based Office of The Affiliated Hospital to Changchun University of Chinese Medicine for their support.

## Author contributions

**Conceptualization:** Meixi Liu, Shoulin Zhang, Xing Liao.

**Data curation:** Meixi Liu, Tianying Chang, Di Zou, ChengJi Cui, Chunyan Liu.

**Formal analysis:** Meixi Liu, Di Zou, ChengJi Cui.

**Funding acquisition:** Shoulin Zhang.

**Investigation:** Shoulin Zhang.

**Methodology:** Meixi Liu, Tianying Chang, Xing Liao.

**Project administration:** Shoulin Zhang, Xing Liao.

**Software:** Meixi Liu, Tianying Chang, Chunyan Liu.

**Supervision:** Shoulin Zhang, Xing Liao.

**Validation:** Tianying Chang, Di Zou, Xing Liao.

**Visualization:** Shoulin Zhang, Tianying Chang, Xing Liao.

**Writing – original draft:** Meixi Liu.

**Writing – review & editing:** Meixi Liu, Tianying Chang, Di Zou, Xing Liao.

## Supplementary Material
