# Supplementary material for: Effects and safety of Ophiocordyceps sinensis preparation in the adjuvant treatment for dialysis patients: A protocol for systematic review and meta-analysis
Source: Medicine (Baltimore). 2022 Nov 18;101(46):e31476. doi: 10.1097/MD.0000000000031476 (PMC9678542; doi:10.1097/MD.0000000000031476)
Supplement: Supplementary file 2 [file medi-101-e31476-s002.pdf]

Search terms

Renal Dialysis（Mesh）
Dialyses, Renal
Renal Dialyses
Dialysis, Renal
Hemodialysis
Hemodialyses
Dialysis, Extracorporeal
Dialyses, Extracorporeal
Extracorporeal Dialyses
Extracorporeal Dialysis
Peritoneal Dialysis（Mesh）
Dialyses, Peritoneal
Dialysis, Peritoneal
Peritoneal Dialyses
Renal Replacement Therapy（Mesh）
Therapy, Kidney Replacement
Replacement Therapy, Renal
Renal Replacement Therapies
Replacement Therapies, Renal
Therapies, Renal Replacement
Therapy, Renal Replacement
Replacement Therapy, Kidney
Kidney Replacement Therapies
Replacement Therapies, Kidney
Therapies, Kidney Replacement
Kidney Replacement Therapy
cordyceps sinensis （MESH）
Ophiocordyceps sinensis
Sphaeria sinensis
Cordyceps sinensis
Cordyceps militaris
Bailing
Jinshuibao
Zhiling
Cordyceps militaris capsules
Yongchongcaojunfen capsules
Cordyceps militaris powder
Yongchongcaojunfen
Cultured cordyceps sinensis poder
Cordyceps Mortierella Mycelia
Cordyceps sinensis powder
Zhiling mycelium
Cordyceps mycelium powder

Cordyceps Cephalosporium Mycelia
Fermented powder of Cephalosporium sinensis
Cordyceps Cephalosporium Mycelia
CePhalosPorium sinensis
Mycelia of Cephalosporium sinensis
fermented Cordyceps powder
Fermented Cordyceps Sinensis powder
Cs-4
Fermentative Cordycepic Fungal Powder
Artificial fermented Cordyceps powder
fermented Paecilomyces hepiali powder
Fermented mycelium of Cordyceps sinensis
Cultured Cordyceps militaris
Cordyceps sinensis fermented mycelium
CsCQ80
Powdred Cordyceps Mortierella Mycelia
ferment powder caterpillar fungus

Chinese Search terms:

1、透析、血液透析、腹膜透析、肾替代疗法、血滤、血透、腹透。

2、虫草、冬虫夏草、人工冬虫夏草制剂、人工虫草制剂、虫草制剂、百令片（胶囊）、金水宝片（胶囊）、至灵胶囊、蛹虫草菌粉胶囊、蛹虫草菌粉、人工虫草菌丝粉、虫草被孢菌粉、至灵菌丝、虫草头孢菌粉、发酵虫草菌粉。

3、全文、随机。

The search strategy for PubMed (record number:114)	
**Number**	**Search terms**
#1	Renal Dialysis[MeSH Terms]
#2	((((((((Renal Dialysis[Title/Abstract]) OR (Dialyses, Renal[Title/Abstract])) OR (Renal Dialyses[Title/Abstract])) OR (Dialysis, Renal[Title/Abstract])) OR (Hemodialysis[Title/Abstract])) OR (Hemodialyses[Title/Abstract])) OR (Dialysis, Extracorporeal[Title/Abstract])) OR (Dialyses, Extracorporeal[Title/Abstract])) OR (Extracorporeal Dialyses[Title/Abstract])) OR (Extracorporeal Dialysis[Title/Abstract])
#3	#1 OR #2
#4	Peritoneal Dialysis[MeSH Terms]
#5	((((Peritoneal Dialysis[Title/Abstract])) OR (Dialyses, Peritoneal[Title/Abstract])) OR (Dialysis, Peritoneal[Title/Abstract])) OR (Peritoneal Dialyses[Title/Abstract])
#6	#4 OR #5
#7	Renal Replacement Therapy[MeSH Terms]
#8	((((((((((Renal Replacement Therapy[Title/Abstract]) OR (Therapy, Kidney Replacement[Title/Abstract])) OR (Replacement Therapy, Renal[Title/Abstract])) OR (Renal Replacement Therapies[Title/Abstract])) OR (Replacement Therapies, Renal[Title/Abstract])) OR (Therapies, Renal Replacement[Title/Abstract])) OR (Therapy, Renal Replacement[Title/Abstract])) OR (Replacement Therapy, Kidney[Title/Abstract])) OR (Kidney Replacement Therapies[Title/Abstract])) OR

	(Replacement Therapies, Kidney[Title/Abstract])) OR (Therapies, Kidney Replacement[Title/Abstract])) OR (Kidney Replacement Therapy[Title/Abstract])
#9	#7 OR #8
#10	#3 OR #6 OR #9
#11	cordyceps sinensis[MeSH Terms]
#12	((((((((((((((((((((((((((((((((cordyceps sinensis[Title/Abstract]) OR (Ophiocordyceps sinensis[Title/Abstract])) OR (Sphaeria sinensis[Title/Abstract])) OR (Cordyceps sinensis[Title/Abstract])) OR (Cordyceps militaris[Title/Abstract])) OR (Bailing[Title/Abstract])) OR (Jinshuibao[Title/Abstract])) OR (Zhiling[Title/Abstract])) OR (Cordyceps militaris capsules[Title/Abstract])) OR (Yongchongcaojunfen capsules[Title/Abstract])) OR (Cordyceps militaris powder[Title/Abstract])) OR (Yongchongcaojunfen[Title/Abstract])) OR (Cultured cordyceps sinensis poder[Title/Abstract])) OR (Cordyceps Mortierella Mycelia[Title/Abstract])) OR (Cordyceps sinensis powder[Title/Abstract])) OR (Zhiling mycelium[Title/Abstract])) OR (Cordyceps mycelium powder[Title/Abstract])) OR (Cordyceps Cephalosporium Mycelia[Title/Abstract])) OR (Fermented powder of Cephalosporium sinensis[Title/Abstract])) OR (Cordyceps Cephalosporium Mycelia[Title/Abstract])) OR (CePhalosPorium sinensis[Title/Abstract])) OR (Mycelia of Cephalosporium sinensis[Title/Abstract])) OR (fermented Cordyceps powder[Title/Abstract])) OR (Fermented Cordyceps Sinensis powder[Title/Abstract])) OR (Cs-4[Title/Abstract])) OR (Fermentative Cordycepic Fungal Powder[Title/Abstract])) OR (Artificial fermented Cordyceps powder[Title/Abstract])) OR (fermented Paecilomyces hepiali powder[Title/Abstract])) OR (Fermented mycelium of Cordyceps sinensis [Title/Abstract])) OR (Cultured Cordyceps militaris[Title/Abstract])) OR (Cordyceps sinensis fermented mycelium[Title/Abstract])) OR (CsCQ80[Title/Abstract])) OR (ferment powder caterpillar fungus[Title/Abstract])) OR (Powdred Cordyceps Mortierella Mycelia[Title/Abstract])) OR (Fermentative Cordycepis Fungal Powder[Title/Abstract])
#13	#11 OR #12
#14	(randomized controlled trial[pt] OR randomized[tiab] OR placebo[tiab] OR drug therapy[sh] OR randomly[tiab] OR trial[tiab] OR groups[tiab] NOT (animals [mh] NOT humans [mh]))
#15	#10 AND #13 AND #14
**The search strategy for Cochrane(record number: 13)**	
#1	MeSH descriptor: [Renal Dialysis] explode all trees 5570
#2	Renal Dialysis 13858
#3	Dialyses, Renal 109
#4	Renal Dialyses 109
#5	Dialysis, Renal 13858
#6	Hemodialysis 13478
#7	Hemodialyses 47
#8	Dialysis, Extracorporeal 392

#9	Dialyses, Extracorporeal 11
#10	Extracorporeal Dialyses 11
#11	Extracorporeal Dialysis 392
#12	#1 OR #2 OR #3 OR #4 OR #5 OR #6 OR #7 OR #8 OR #9 OR #10 OR #11 20628
#13	MeSH descriptor: [Peritoneal Dialysis] explode all trees 924
#14	Peritoneal Dialysis 2546
#15	Dialyses, Peritoneal 24
#16	Dialysis, Peritoneal 2546
#17	Peritoneal Dialyses 24
#18	#13 OR #14 OR #15 OR #16 OR #17 2546
#19	MeSH descriptor: [Renal Replacement Therapy] explode all trees 9691
#20	Renal Replacement Therapy 3823
#21	Therapy, Kidney Replacement 2756
#22	Replacement Therapy, Renal 3823
#23	Renal Replacement Therapies 401
#24	Replacement Therapies, Renal 401
#25	Therapies, Renal Replacement 401
#26	Therapy, Renal Replacement 3823
#27	Replacement Therapy, Kidney 2756
#28	Kidney Replacement Therapies 302
#29	Replacement Therapies, Kidney 302
#30	Therapies, Kidney Replacement 302
#31	Kidney Replacement Therapy 2756
#32	#19 OR #20 OR #21 OR #22 OR #23 OR #24 OR #25 OR #26 OR #27 OR #28 OR #29 OR #30 OR #31 13195
#33	#12 OR #18 OR #32 27142
#34	MeSH descriptor: [Cordyceps] explode all trees 25
#35	cordyceps sinensis 60
#36	Ophiocordyceps sinensis 16
#37	Sphaeria sinensis 1
#38	Cordyceps sinensis 60
#39	Cordyceps militaris 15
#40	Bailing 37
#41	Jinshuibao 28
#42	Zhiling 7
#43	Cordyceps militaris capsules 5
#44	Yongchongcaojunfen capsules 0
#45	Cordyceps militaris powder 1
#46	Yongchongcaojunfen 0
#47	Cultured cordyceps sinensis poder 1
#48	Cordyceps Mortierella Mycelia 0
#49	Cordyceps sinensis powder 8
#50	Zhiling mycelium 0

#51	Cordyceps mycelium powder 1
#52	Cordyceps Cephalosporium Mycelia 0
#53	Fermented powder of Cephalosporium sinensis 0
#54	Cordyceps Cephalosporium Mycelia 0
#55	CePhalosPorium sinensis 0
#56	Mycelia of Cephalosporium sinensis 0
#57	fermented Cordyceps powder 4
#58	Fermented Cordyceps Sinensis powder 3
#59	Cs-4 49
#60	Fermentative Cordycepic Fungal Powder 0
#61	Artificial fermented Cordyceps powder 0
#62	fermented Paecilomyces hepiali powder 0
#63	Fermented mycelium of Cordyceps sinensis 1
#64	Cultured Cordyceps militaris 1
#65	Cordyceps sinensis fermented mycelium 1
#66	CsCQ80 0
#67	ferment powder caterpillar fungus 0
#68	Powdred Cordyceps Mortierella Mycelia 0
#69	Fermentative Cordycepis Fungal Powder 0
#70	#34 OR #35 OR #36 OR #37 OR #38 OR #39 OR #40 OR #41 OR #42 OR #43 OR #44 OR #45 OR #46 OR #47 OR #48 OR #49 OR #50 OR #51 OR #52 OR #53 OR #54 OR #55 OR #56 OR #57 OR #58 OR #59 OR #60 OR #61 OR #62 OR #63 OR #64 OR #65 OR #66 OR #67 OR #68 OR #69 186
#71	randomized controlled trial 1144828
#72	randomized 1269054
#73	placebo 354202
#74	drug therapy 483856
#75	randomly 287465
#76	trial 1406316
#77	groups 543257
#78	MeSH descriptor: [Animation] explode all trees 0
#79	MeSH descriptor: [Humanism] explode all trees 14
#80	#71 OR #72 OR #73 OR #74 OR #75 OR #76 OR #77 1617518
#81	#78 NOT #79 0
#82	#80 NOT #81 1617518
#83	#33 AND #70 AND #82 13
**The search strategy for Embase (record number: 4)**	
#1	'hemodialysis'/exp 128,169
#2	'renal dialysis':ti,ab,kw OR 'dialyses, renal':ti,ab,kw OR 'renal dialyses':ti,ab,kw OR 'dialysis, renal':ti,ab,kw OR hemodialysis:ti,ab,kw OR hemodialyses:ti,ab,kw OR 'dialysis, extracorporeal':ti,ab,kw OR 'dialyses,extracorporeal':ti,ab,kw OR 'extracorporeal dialyses':ti,ab,kw OR 'extracorporeal dialysis':ti,ab,kw

	107,400
#3	#1 OR #2 164,476
#4	'peritoneal dialysis'/exp 48,171
#5	'peritoneal dialysis':ti,ab,kw OR 'dialyses, peritoneal':ti,ab,kw OR 'dialysis,peritoneal':ti,ab,kw OR 'peritoneal dialyses':ti,ab,kw 37,964
#6	#4 OR #5 52,726
#7	'renal replacement therapy'/exp 222,870
#8	'renal replacement therapy':ti,ab,kw OR 'therapy,kidney replacement':ti,ab,kw OR 'replacement therapy, renal':ti,ab,kw OR 'renal replacement therapies':ti,ab,kw OR 'replacement therapies, renal':ti,ab,kw OR 'therapies, renal replacement':ti,ab,kw OR 'therapy, renal replacement':ti,ab,kw OR 'replacement therapy, kidney':ti,ab,kw OR 'kidney replacement therapies':ti,ab,kw OR 'replacement therapies,kidney':ti,ab,kw OR 'therapies, kidney replacement':ti,ab,kw OR 'kidney replacement therapy':ti,ab,kw 29,067
#9	#7 OR #8 226,708
#10	#3 OR #6 OR #9 250,440
#11	'ophiocordyceps sinensis'/exp 193
#12	'ophiocordyceps sinensis':ti,ab,kw OR 'sphaeria sinensis':ti,ab,kw OR 'cordyceps sinensis':ti,ab,kw OR 'cordyceps militaris':ti,ab,kw OR bailing:ti,ab,kw OR jinshuibao:ti,ab,kw OR zhiling:ti,ab,kw OR 'cordyceps militaris capsules':ti,ab,kw OR 'yongchongcaojunfen capsules':ti,ab,kw OR 'cordyceps militaris powder':ti,ab,kw OR yongchongcaojunfen:ti,ab,kw OR 'cultured cordyceps sinensis poder':ti,ab,kw OR 'cordyceps mortierella mycelia':ti,ab,kw OR 'cordyceps sinensis powder':ti,ab,kw OR 'zhiling mycelium':ti,ab,kw OR 'cordyceps mycelium powder':ti,ab,kw OR 'fermented powder of cephalosporium sinensis':ti,ab,kw OR 'cordyceps cephalosporium mycelia':ti,ab,kw OR 'cephalosporium sinensis':ti,ab,kw OR 'mycelia of cephalosporium sinensis':ti,ab,kw OR 'fermented cordyceps powder':ti,ab,kw OR 'fermented cordyceps sinensis powder':ti,ab,kw OR 'cs4':ti,ab,kw OR 'fermentative cordycepic fungal powder':ti,ab,kw OR 'artificial fermented cordyceps powder':ti,ab,kw OR 'fermented paecilomyces hepiali powder':ti,ab,kw OR 'fermented mycelium of cordyceps sinensis':ti,ab,kw OR 'cultured cordyceps militaris':ti,ab,kw OR 'cordyceps sinensis fermented mycelium':ti,ab,kw OR cscq80:ti,ab,kw OR 'ferment powder caterpillar fungus':ti,ab,kw OR 'powdred cordyceps mortierella mycelia':ti,ab,kw OR 'fermentative cordycepis fungal powder':ti,ab,kw 2,435
#13	#11 OR #12 2,475
#14	'crossover procedure':de OR 'double-blind procedure':de OR 'randomized controlled trial':de OR 'single-blind procedure':de OR random*:de,ab,ti OR factorial*:de,ab,ti OR crossover*:de,ab,ti OR ((cross NEXT/1 over*):de,ab,ti) OR placebo*:de,ab,ti OR ((doubl*NEAR/1 blind*):de,ab,ti) OR ((singl* NEAR/1 blind*):de,ab,ti) OR assign*:de,ab,ti OR allocat*:de,ab,ti OR volunteer*:de,ab,ti 2,969,612
#15	#10 AND #13 AND #14 4
**The search strategy for CLNICALTRAILS(record number: 11)**	

cordyceps sinensis OR Ophiocordyceps sinensis OR Sphaeria sinensis OR Cordyceps sinensis OR Cordyceps militaris OR Bailing OR Jinshuibao OR Zhiling OR Cordyceps militaris capsules OR Yongchongcaojunfen capsules OR Cordyceps militaris powder OR Yongchongcaojunfen OR Cultured cordyceps sinensis poder OR Cordyceps Mortierella Mycelia OR Cordyceps sinensis powder OR Zhiling mycelium OR Cordyceps mycelium powder OR Cordyceps Cephalosporium Mycelia OR Fermented powder of Cephalosporium sinensis OR Cordyceps Cephalosporium Mycelia OR CePhalosPorium sinensis OR Mycelia of Cephalosporium sinensis OR fermented Cordyceps powder OR Fermented Cordyceps Sinensis powder OR Cs-4 OR Fermentative Cordycepic Fungal Powder OR Artificial fermented Cordyceps powder OR fermented Paecilomyces hepiali powder OR Fermented mycelium of Cordyceps sinensis OR Cultured Cordyceps militaris OR Cordyceps sinensis fermented mycelium OR CsCQ80 OR Powdred Cordyceps Mortierella Mycelia OR ferment powder caterpillar fungus
**The search strategy for ICTRP(record number: 33)**
cordyceps sinensis OR Ophiocordyceps sinensis OR Sphaeria sinensis OR Cordyceps sinensis OR Cordyceps militaris OR Bailing OR Jinshuibao OR Zhiling OR Cordyceps militaris capsules OR Yongchongcaojunfen capsules OR Cordyceps militaris powder OR Yongchongcaojunfen OR Cultured cordyceps sinensis poder OR Cordyceps Mortierella Mycelia OR Cordyceps sinensis powder OR Zhiling mycelium OR Cordyceps mycelium powder OR Cordyceps Cephalosporium Mycelia OR Fermented powder of Cephalosporium sinensis OR Cordyceps Cephalosporium Mycelia OR CePhalosPorium sinensis OR Mycelia of Cephalosporium sinensis OR fermented Cordyceps powder OR Fermented Cordyceps Sinensis powder OR Cs-4 OR Fermentative Cordycepic Fungal Powder OR Artificial fermented Cordyceps powder OR fermented Paecilomyces hepiali powder OR Fermented mycelium of Cordyceps sinensis OR Cultured Cordyceps militaris OR Cordyceps sinensis fermented mycelium OR CsCQ80 OR Powdred Cordyceps Mortierella Mycelia OR ferment powder caterpillar fungus
**The search strategy for CNKI (record number: 133)**
(TKA='透析' or TKA='血液透析' or TKA='腹膜透析' or TKA='肾替代疗法' or TKA'血滤' or TKA'血透' or TKA'腹透') AND (TKA='虫草' or TKA='冬虫夏草' or TKA='人工冬虫夏草制剂' or TKA='人工虫草制剂' or TKA='虫草制剂' or TKA='金水宝胶囊' or TKA='金水宝片' or TKA='百令胶囊' or TKA='百令片' or TKA='至灵胶囊' or TKA='蛹虫草菌粉胶囊' or TKA='蛹虫草菌粉' or TKA='人工虫草菌丝粉' or TKA='虫草被孢菌粉' or TKA='至灵菌丝' or TKA='虫草头孢菌粉' or TKA='发酵虫草菌粉') AND (FT='随机')
**The search strategy for Wanfang (record number: 189)**
(题名或关键词=(“透析” or “血液透析” or “腹膜透析” or “肾替代疗法” or “血滤” or “血透” or “腹透”)) AND (“虫草” or “冬虫夏草” or “人工冬虫夏草制剂” or “人工虫草制剂” or “虫草制剂” or “金水宝胶囊” or “金水宝片” or “百令胶囊” or “百令片” or “至灵胶囊” or “蛹虫草菌粉胶囊” or “蛹虫草菌粉” or “人工虫草菌丝粉” or “虫草被孢菌粉” or “至灵菌丝” or “虫草头孢菌粉” or “发酵虫草菌粉”)) AND (全文= (“随机”))

The search strategy for VIP (record number: 70)
(M=(透析 or 血液透析 or 腹膜透析 or 肾替代疗法 or 血滤 or 血透 or 腹透)) AND M=(虫草 or 冬虫夏草 or 人工冬虫夏草制剂 or 人工虫草制剂 or 虫草制剂 or 金水宝胶囊 or 金水宝片 or 百令胶囊 or 百令片 or 至灵胶囊 or 蛹虫草菌粉胶囊 or 蛹虫草菌粉 or 人工虫草菌丝粉 or 虫草被孢菌粉 or 至灵菌丝 or 虫草头孢菌粉 or 发酵虫草菌粉 or 虫草头孢菌粉胶囊) AND U=随机
**The search strategy for SinoMed (record number: 100)**
1) "透析"[核心字段:智能] OR "血液透析"[核心字段:智能] OR "腹膜透析"[核心字段:智能] OR "肾替代疗法"[核心字段:智能] OR "血滤"[核心字段:智能] OR "血透"[核心字段:智能] OR "腹透"[核心字段:智能] 98424<br>2) "虫草"[核心字段:智能] OR "冬虫夏草"[核心字段:智能] OR "人工冬虫夏草制剂"[核心字段:智能] OR "人工虫草制剂"[核心字段:智能] OR "虫草制剂"[核心字段:智能] OR "百令"[核心字段:智能] OR "金水宝"[核心字段:智能] OR "至灵胶囊"[核心字段:智能] OR "蛹虫草菌粉胶囊"[核心字段:智能] OR "蛹虫草菌粉"[核心字段:智能] OR "人工虫草菌丝粉"[核心字段:智能] OR "虫草被孢菌粉"[核心字段:智能] OR "至灵菌丝"[核心字段:智能] OR "虫草头孢菌粉"[核心字段:智能] OR "发酵虫草菌粉"[核心字段:智能] 6462<br>3) "随机"[全部字段:智能] 1755212<br>4) (#3) AND (#2) AND (#1) 100
